# Belladonna in Incontinence of Urine

**Published:** 1857-08

**Authors:** 


*Belladonna in Incontinence of Urine.*—In the British Medical Journal a case is reported of incontinence of urine in a child eight years of age, which had existed since its birth. She had been treated with purgatives alkalies, blisters to sacrum, &c., without improvement. She was finally relieved by the use of $\frac{1}{8}$ of a grain of Ext. Belladonna *night and morning.*

